# Who Are We Feeding? Asymmetric Individual Use of Surplus Food Resources in an Insular Population of the Endangered Egyptian Vulture *Neophron percnopterus*

**DOI:** 10.1371/journal.pone.0080523

**Published:** 2013-11-11

**Authors:** Marie-Sophie García-Heras, Ainara Cortés-Avizanda, José-Antonio Donázar

**Affiliations:** 1 Department of Conservation Biology, Estación Biológica de Doñana C.S.I.C., Sevilla, Spain; 2 Percy FitzPatrick Institute of African Ornithology, DST/NRF Centre of Excellence, University of Cape Town, Rondebosch, South Africa; 3 Theoretical Ecology and Biodiversity Change Group, Centro de Biologia Ambiental, Faculdade de Ciências da Universidade de Lisboa, Lisbon, Portugal; University of Jyväskylä, Finland

## Abstract

Supplementary feeding stations, or “vulture restaurants”, are common conservation management tools. While a number of studies have investigated the consequences of surplus food on the population dynamics of scavengers, relatively little is known about the effects of such practices at the individual level. Within the long-term monitored breeding population of Canarian Egyptian vultures (*Neophron percnopterus majorensis*) we investigated individual bird’s patterns of use of a supplementary feeding station at Fuerteventura (Canary Islands), over the course of breeding (2001, 2002; 2004-2011) and non-breeding seasons (2000-2010). Our results show that during the breeding season the individual use of the supplementary feeding station was inversely related to the distance to the breeding territory, which suggests the existence of central-place foraging constraints. In addition, larger birds of poor body-condition and individuals that ultimately failed to fledge young were detected more frequently at the feeding station. During the non-breeding season, and because most breeding birds abandoned the breeding territories, the overall abundance of Egyptian vultures at the feeding station grew. Moreover, the only variable increasing the probability of presence of individuals was poor body condition so that birds with lower wing residual visited the feeding station more frequently. Supplementary feeding may benefit individuals who would otherwise have been subject to selective pressures. From our results it follows that this conservation strategy must be used with caution because it can have consequences on an individual level and thus potentially affect the viability of endangered populations.

## Introduction

Almost every area of an animal’s ecology, from individual behaviour, survival, or demography to population distribution, may be affected by changes in food availability [1-8]. The foraging activities of wild animals are shaped by the spatial distribution of trophic resources, which may be profoundly modified in space and time by human activities, inducing ecological consequences for populations [1,9-11]. To counteract these negative effects, supplementary feeding has become a widely used conservation tool to achieve rapid recovery of endangered taxa [5,7,12-16]. This is particularly true for threatened scavengers. The provision of surplus food at supplementary feeding stations (SFS), or so-called “vulture restaurants”, is a worldwide practice to facilitate the recovery of populations of these birds [9,17]. At those sites, food is provided to counter both the scarcity of natural food sources and illegal poisoning with the hope of improving demographic parameters and ultimately population viability [18].

The effects of supplementary feeding programs on populations have received considerable attention [9,16]. An increasing number of studies have reported that the SFS may increase breeding output, individual survival and territory persistence [16,19-21]. Conversely, supplementary feeding favors the aggregation of non-breeders (floaters), which have been demonstrated to induce negative density-dependent processes affecting reproductive success [22]. Moreover, the predictability inherent in the SFS favors dominant and abundant species that monopolize food to the detriment of other less competitive scavengers of higher conservation interest [9]. Finally, the effects of food predictability may permeate through other trophic levels even affecting primary consumers through predation risk derived from facultative predator-scavengers that concentrate near SFS [23,24]. However, there is almost a total lack of knowledge on the effects that these practices may have at the individual level. Since populations are formed by individuals with different responses to similar environmental pressures, we could expect that the use of SFS would promote changes in selective pressures related to the use of trophic resources [4]. Thus, a deeper understanding of those responses is necessary in order to optimize the management of supplementary feeding to enhance the viability of threatened populations.

By becoming predictable in space, supplementary feeding may impose significant constraints from the point of view of foraging ecology and individual decisions. Many breeding birds exhibiting a central-place foraging behaviour, which leads them to exploit space differentially, depending on the time and energy expenditure involved in hauling prey to the nest [25]. Thus, only those individuals breeding in close vicinity to the feeding place would benefit from additional trophic contributions. Apart from this, it is well known that the individuals differ not only in relation to physical characteristics such as size and body condition, which in turn may determine the possibility of exploiting these surplus food resources where the degree of competition may be high (see e.g. 26), but also in personality traits (correlated behavioural tendencies forming syndromes) thus determining, the exploration-avoidance of the novel food resources [27,28].

Here we study the individual use of a supplementary feeding station by an insular Egyptian Vulture (*Neophron percnopterus*) population endemic to the Canary archipelago [29-31]. We took advantage of a long-term intensive monitoring program that has allowed the individual identification of 85% of the birds. Particularly, we test to what extent the presence at SFS of adult territorial vultures was determined by extrinsic (environmental) and/or intrinsic (individual) factors. Among the first group of variables, we paid special attention to the distance from the SFS to the breeding territory. Specifically and because of central-place foraging constraints we predict an inverse relationship between them. Within the second group of variables we looked particularly at the relationship between the use of the SFS and variables evaluating individual quality. We predict that parents in inferior condition may depend more heavily upon the supplementary food [4,5,32].

## Materials and Methods

### Ethics statement

The entire study was observational and did not require approval of the animal ethics committee. Visits to the feeding station were done within a research-monitoring program in collaboration with the Cabildo Insular of Fuerteventura which has the competences to regulate observation and research on wildlife and endangered species.

### Focal species and study area

**Figure 1:**
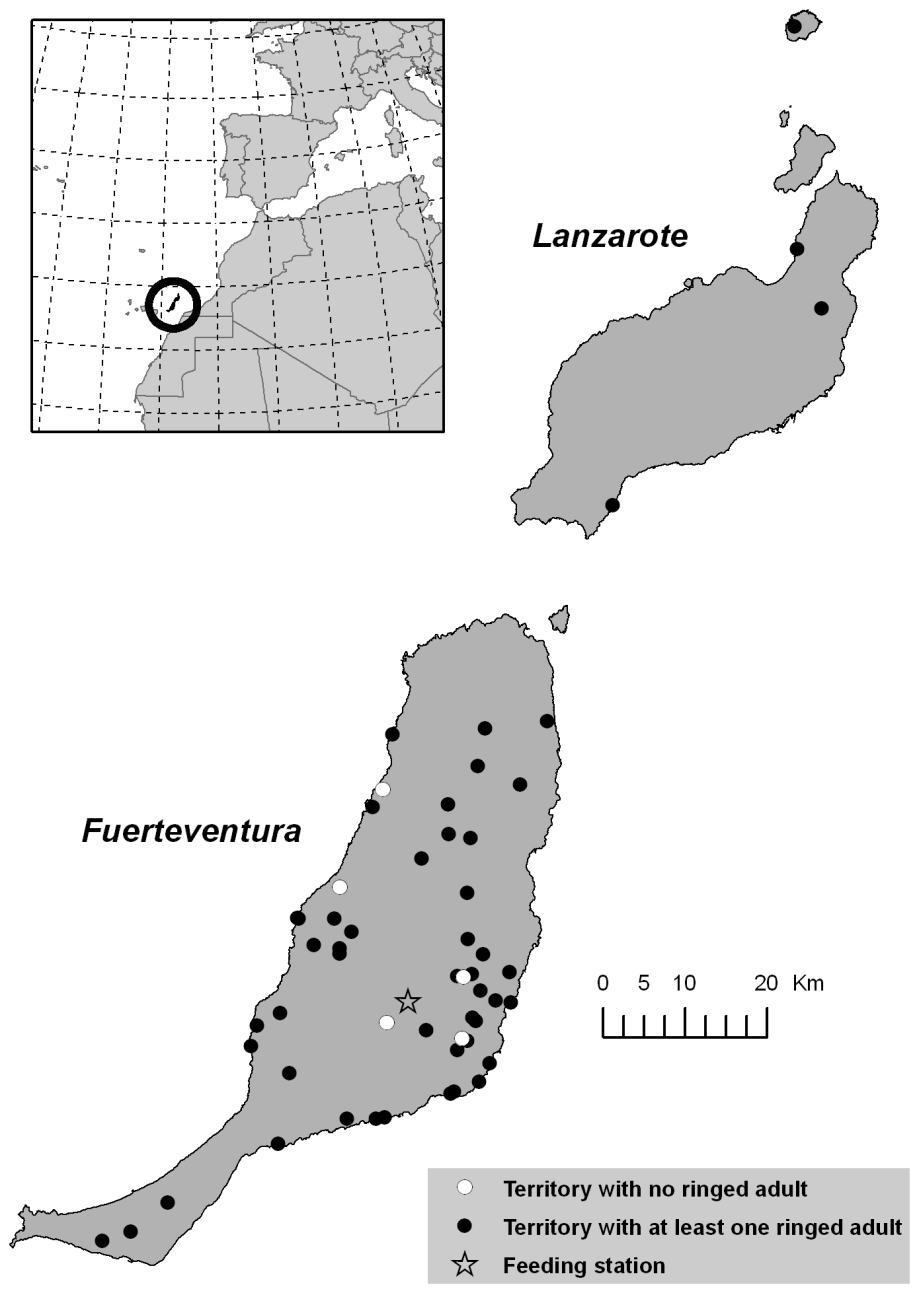
Study area and active Egyptian vulture territories between 2000-2011. Territories with both unringed and at least one ringed breeding adult are shown by white and black circles, respectively. The star denotes the position of the supplementary feeding station.

The Canarian Egyptian vulture (N. p. *majorensis*) is a sedentary and endemic threatened long-lived bird of prey having undergone a severe decline during the 20th century, leading to its classification as “critically endangered” [29-31,33-35]. Fuerteventura is the stronghold of the population, although some individuals are also present on Lanzarote Island and adjacent islets (Figure 1). Long-term intensive monitoring of this population began in 1998 when only 23 territories were occupied. The population has subsequently increased to 50 territories in 2011 (total population size N=200). Individuals were captured at the nest (fledglings) or by means of cannon-nets in the areas surrounding the SFS (i.e., immatures and adults). All were ringed with metal and plastic colour rings with alphanumeric codes permitting identification from a distance. During our study period (2000-2011) a total of 170 individuals were ringed. The number of occupied territories per year was variable: a total number of 56 different territories of Egyptian vultures were located during the study period; of these 50 had at least one ringed breeder. The annual number varied between 3 (2000) to 39 (2011) (see Figure 1 for the distribution of the breeding territories during the last year). A total of 102 breeding birds (45 males and 57 females) were monitored during the study period. 

A SFS was implemented in 1998 by local authorities as a management tool for the conservation of this endangered population. This site is supplied regularly with goat (*Capra hircus*) and pig (*Sus scrofa* var. dom*.*) carcasses (1-4 per week) and slaughterhouse remains (c. 200 kg per week). There, Egyptian vultures congregate in large numbers (up to 130 different individuals in a single day), especially during the non-breeding season, together with large numbers of other facultative scavenger species such as common buzzards (*Buteo buteo*) and common ravens (*Corvux corax*) [36]. Outside of the SFS, the Egyptian vultures consume randomly encountered carcasses, especially those of goats and small-sized vertebrates, mainly wild rabbits (*Oryctolagus cuniculus*) and birds [33,37].

### Field procedures

The presence of territorial Egyptian vultures at the SFS was monitored during the breeding period (January-June) in 2001, 2002, and 2004-2011 (n=119 days, 5-23 days/year) and the non-breeding period (July-December) in 2000-2010 (n=210 days, 5-41 days/year).

Observations were concentrated on those days when food was supplied. All the observations were conducted under good weather conditions. The observer was in a hidden area 15m from the food, and the birds showed no reaction to this structure. Observations began when the food was deposited (usually around 9.00-10.00 a.m.) and finished at dusk. Plastic rings were read by means of telescopes (20-60X). 

### Analytical procedures

Generalized linear mixed models (GLMM) [38] were fitted independently for data collected in both breeding and non-breeding seasons. We used a binomial distribution of errors and a logistic link function, testing for significance of each variable by using F tests. Instead of using a single figure representing the proportion of days that each individual was present, we modelled the response variable as the number of days present with the total number of observation days as a binomial denominator (see e.g. 39). By doing so, more relevance was given to proportions coming from larger sample sizes [40].

In the two trials of analyses we fitted ten explanatory variables. Three of them (1), individual (2), year and (3) territory, were fitted as random terms to avoid non-independence of data because of inter-annual variability. The following variables (4), distance (km) between the territory and the feeding station and (5) island (a two-level factor: Fuerteventura and Lanzarote), evaluated environmental features inherent to the breeding areas and their spatial position in relation to the SFS. Another three variables described intrinsic individual factors: (6) sex, (7) wing chord (cm between the carpal joint of the bent wing and the tip of the longest straightened primary [41,42]) and (8) body condition, estimated from wing residuals (linear regression between body mass and wing chord, taking into account the sex of each individual [31,43,44]). We also examined the relationship to (9) breeding success (presence or absence of fledglings in the nest). Finally (10) we considered the reproductive stage distinguishing between those observations carried out during courtship and incubation (“early” stage: January-April) and chick rearing (“late” stage: May-July). Modeling was performed using a forward stepwise procedure [9,45], with the SAS 9.2. program [38,46]. Macro GLIMMIX were automatically adjusts for over dispersion [38]. All tests were two tailed.

## Results

Between 2000-2011, 45.3% and 77.5% of the territorial ringed Egyptian vultures (N=102) visited the SFS during the breeding and non-breeding seasons, respectively, with the rates being clearly higher during the non-reproductive period (Table 1). In addition, there were large variations between individuals (0-100%) in the rate of visits, females using the station slightly more than males.

**Table 1:** Summary of the individual rates of visits (%) of territorial ringed Egyptian vultures, to the supplementary feeding station between 2000-2011.

	**Breeding**								**Non-breeding**			
	Early (Jan-Apr)				Late (May-Jul)							
	Mean	s.d.	range	N^a^	Mean	s.d.	range	N^a^	Mean	s.d.	range	N^a^
Males	20.8	21.0	0-92	41	11.8	20.4	0-89	39	31.8	23.6	0-75	34
Females	25.7	21.1	0-81	56	14.6	21.3	0-83	54	38.1	25.2	0-100	50

	**Mean**		**s.d.**		**range**		**N^a^**		**Mean**	**s.d.**	**range**	**N^a^**
Total	18.6		21.6		0-91		97		36.0	24.6	0-100	84

^a^number of territorial Egyptian vultures monitored

Modelling procedures showed that during the breeding season the probability of presence of territorial vultures at the SFS was higher for those birds breeding at closer distances, of greater size (wing chord) and showing more negative values for body condition (wing-weight residuals) (Table 2). In addition, visits were more probable during the early stage of the reproductive cycle and for those birds that ultimately failed to fledge young. Finally, it was very remarkable that although the SFS was placed in Fuerteventura, vultures breeding on this island were globally less frequently detected than those holding territories in Lanzarote. During the non-breeding season the presence of birds at the feeding site was only related (inversely) to the wing-weight residual.

**Table 2:** Summary of the results from the GLMMs explaining the frequency of days on which the feeding station was visited by individual Egyptian vultures.

**Season/Effect**	**Estimate**	**SE**	***F*-value(df)**	***P***
Breeding				
Intercept	-5.204	1.412	-3.69^a^ (9)	0.0050
Wing	0.006	0.003	5.22 (1,438)	0.0228
Body condition	-6.588	2.546	6.69 (1,438)	0.0100
Distance	-0.023	0.008	7.34 (1,438)	0.0070
Island^b^	-0.932	0.301	9.58 (1,438)	0.0021
Breeding success^c^	0.405	0.132	9.34 (1,438)	0.0024
Stage^d^	1.605	0.266	26.43 (1,438)	<0.0001
Individual^e^	0.301	0.105		
Year^e^	0.352	0.102		
Territory^e^	0.778	0.258		
Non-breeding				
Intercept	0.759	0.265	2.87^a^ (10)	0.0168
Body condition	-3.975	1.896	4.39 (1,240)	0.0373
Individual^e^	0.840	0.261		
Year^e^	0.263	0.122		
Territory^e^	1.067	0.420		

Number of visits to the feeding station/Number of controls (in days) was the response variable. Estimated effects, SE (standard error), F-values, df (degrees of freedom) and associated probabilities are shown for those variables significantly improving the fitting of the models.

^a^t-test

Estimates correspond to: ^b^Fuerteventura Island; ^c^Breeding failure; ^d^Early breeding stage; levels for Lanzarote Island, positive breeding success and late breeding stage were 0.

^e^Random effect

## Discussion

Our results suggest that variables related to the breeding site (distance to the feeding station, island) as well as those inherent to the individual (size, body condition) and its breeding success are related to the use of a supplementary feeding station during the breeding season. The location of the breeding territory may be determinant because in central-place foragers, the distance to the trophic resource and the breeding site implies costs associated with the time and energy devoted to displacements and food-carrying [25]. In fact, the Canarian Egyptian vulture is, as are many birds of prey, a single-prey loader (i.e., one prey item per food-trip, [47]) having to bring food back frequently to their nests to feed young [48,49]. Satellite radiotelemetry studies carried out in continental regions of Spain also show that trophic resources are obtained mostly within short ranges (up to 8 km) from the nest but occasionally breeding birds can visit places up to 30 km away (unpublished data). In our case, adult birds breeding up to 103 km away have been observed in the SFS, corresponding to a nest placed in the north of Lanzarote Island. These long-distance visits are likely linked to very poor food conditions in the habitually exploited home ranges [50]. The fact that birds holding territories in Lanzarote show, as a whole, higher probabilities of visiting the feeding station despite the long distance to travel instead again indicates that inter-island variability in food conditions are determinant and provides insights about how availability of trophic resources, among other factors, can shape movements between islands and ultimately meta-population dynamics [51]. 

Three other variables significantly related to the probability of presence were size, body condition, and breeding success. The two first variables indicate that individual quality may be decisive in relation to the exploitation of surplus food resources. Thus, large birds with higher competitive abilities [26] but of lower body-condition would be more prone to exploit these predictable resources where food should be shared with conspecifics and other social and aggressive species like common ravens and common buzzards [33]. In regards to the relationship to breeding success, this could be interpreted preliminarily as a consequence of breeding failure: those birds exempt from breeding tasks would visit the station more frequently but the fact that “breeding stage” fit significantly into the model and in an opposite way (higher estimates in early breeding periods) suggests that again this variable reflects that poor-quality birds are more likely to visit the feeding station. This result is also reinforced by the fact that during the late breeding periods, only 47.4% of the territorial birds visited the SFS vs. 77.3% during the early breeding periods.

What proximal factors are behind the relationship between the frequency of visits and breeding success? First, this could be related to individual and/or territory (home range) quality. Quality of breeders may be reflected in better body reserves, foraging skills or nest defense ability [52,53] whereas territory quality may be apparent in the quality, abundance and diversity of trophic resources, safe nest-sites and refuges, and distance to the nearest neighbor [32,42,54-58]. In our case, breeders of higher physiological quality and/or exploiting foraging habitats of better quality would be better able to find unpredictable food in the wild. As was stated above, Egyptian vultures exploit small and medium-sized vertebrate carcasses, which may provide indispensable elements to the diet, such as calcium [22,59-61]. These very arguments may explain why individuals of good body condition are less attached to the feeding station in the winter: they are probably more able to exploit high-quality resources without having to share them with conspecific and interspecific competitors.

The absence of breeding tasks is clearly behind the increase in the presence of adult vultures at the feeding station during the non-breeding season. The fact that the main wild prey of Egyptian vultures (carcasses of small vertebrates like young mammals, birds and reptiles [62]) is scarcer during the cold season may also be at play, driving the birds to exploit predictable resources. Apart from this, it cannot be ruled out that social factors also favor concentrations of birds during the non-breeding seasons. Thus, as has been demonstrated in other bird species, socialization may be an important pressure leading birds to join communal roosts during non-breeding seasons [30,63,64]. Virtually all the Egyptian vultures in our study area gather in a large (>100 individuals) roost [33].

Individual sex had no significant influence but it seems likely that during the breeding season females visited the supplementary feeding station slightly more frequently than males. Egyptian vultures show reversed sexual dimorphism see 29, which could lead to asymmetric foraging strategies and differential use of food resources [65]. Females dominate in contests for food (authors, unpublished data) and may obtain greater benefits from places supplied with large carcasses where many birds congregate. This is corroborated by the lower susceptibility of female Canarian Egyptian vultures to poisoning by lead ammunition; this suggests that they preferentially exploit large ungulate carcasses (found in predictable places) over the remains of small vertebrates [66]. 

To our knowledge, our paper represents the first attempt to examine how individuals of a long-lived scavenger species use surplus food provided for conservation purposes. Supplementary feeding makes the availability of carrion resources predictable (both in space and time), which is otherwise unpredictable in the wild, having consequences at both population and community levels, from passerines to seabirds and scavengers [19-24]. Here, we show how these predictable resources can be used asymmetrically by individual breeding vultures. The long-term population consequences of these facts are unknown. First, because of time and energy constraints, supplementary feeding stations may favor the settlement of breeding individuals within relatively short and medium distances, thus promoting spatial heterogeneity within the population leading to effects like territory shrinkage and productivity depression within the SFS radius of influence [22,67]. In fact, the Egyptian vulture breeding density is maximum in the central area of Fuerteventura Island (80% of the territories are less than 30 km away from the SFS), which may suggest that the SFS has determined local nest bunching. Second, feeding sites seem to be used preferentially by individuals with relatively low breeding performance and body condition. This kind of scenario is purposely promoted by game managers who seek to reduce natural herbivore mortality in order to improve hunting activities [68,69] but has been almost neglected within conservation strategies of threatened vertebrates subject to spatial subsides. The issue is not trivial because this kind of habitat manipulation may be benefiting a fraction of the population that otherwise would be subject to strong selective pressures [4]. Long-term effects of supplementary feeding, therefore, are not limited to the alteration of basic demographic parameters [5,6] but individual-scale qualitative changes can also occur with unknown population viability consequences.
